# Distribution of Private Dental Healthcare Facilities in Riyadh City: A GIS-Based Approach

**DOI:** 10.3390/ijerph21070959

**Published:** 2024-07-22

**Authors:** Najla S. Alrejaye, Faisal H. Alonazi, Zaid M. Alonazi, Rahf S. Alobaidi, Asma B. Alsaleh, Alanoud A. Alshami, Sultan A. Alshamrani, Seena T. Kaithathara

**Affiliations:** 1 Department of Dental Services, King Abdulaziz Medical City, Ministry of National Guard Health Affairs, Riyadh 11426, Saudi Arabia; 2 Department of Preventive Dental Science, College of Dentistry, King Saud bin Abdulaziz University for Health Sciences, Riyadh 11481, Saudi Arabia; 3 King Abdullah International Medical Research Center, Ministry of National Guard Health Affairs, Riyadh 11481, Saudi Arabia; 4 Internship Unit, College of Dentistry, King Saud bin Abdulaziz University for Health Sciences, Riyadh 11481, Saudi Arabia; alenzi461@ksau-hs.edu.sa (F.H.A.); alobaidi290@ksau-hs.edu.sa (R.S.A.); alsaleh012@ksau-hs.edu.sa (A.B.A.); alshami187@ksau-hs.edu.sa (A.A.A.);; 5 Department of Biostatistics and Bioinformatics, King Abdullah International Medical Research Center, King Saud Bin Abdulaziz University for Health Sciences Ministry of National Guard Health Affairs, Riyadh 11481, Saudi Arabia; thomass@kaimrc.edu.sa

**Keywords:** accessibility, location, geographic information system, dental clinics, population, Kingdom of Saudi Arabia

## Abstract

Background: The dental healthcare private sector in Riyadh city has been growing rapidly over the past few years; however, there is a lack of information on the accessibility and spatial distribution of private dental healthcare facilities (PDHFs) in the area. This study aimed to evaluate the spatial distribution of PDHFs in Riyadh city in relation to population density in each sub-municipality. Methods: The current information regarding the number, location, and operability of PDHFs in Riyadh city was obtained from the Ministry of Health. A total of 632 operating PDHFs were included with the precise location plotted on Quantum Geographic Information System software (version 3.32.1, Essen, Germany) using Google Earth. Four levels of buffer zones—1 km, 3 km, 5 km, and >5 km—were determined. The population statistics and mean monthly individual income per district were gathered from Zadd.910ths. Microsoft Excel (version 16.0, Microsoft, Redmond, WA, USA) and RStudio software (version 4.1.3, Posit Software, PBC, Boston, MA, USA) were used for additional data analysis. Results: There was an overall ratio of one PDHF per 9958 residents in Riyadh city. Olaya and Maather sub-municipalities had the largest PDHF-to-population ratios: (1:4566) and (1:4828), respectively. Only 36.3% of the city’s total area was within a 1 km buffer zone from a PDHF. There was an overall weak positive correlation between the number of PDHFs and the total area in each sub-municipality (r = 0.29), and the distribution of PDHFs was uneven corresponding to the area (G* = 0.357). Conclusions: There was an uneven distribution of PDHFs in Riyadh city. Some areas were underserved while others were overserved in several sub-municipalities. Policy-makers and investors are encouraged to target underserved areas rather than areas with significant clustering to improve access to care.

## 1. Introduction

Accessibility is one of the five main characteristics required to achieve optimal access to care. Access to healthcare services is defined as the ability of the population to receive and utilize health services when needed [1], and it reflects a set of more specific dimensions describing the fit between the patient and the health care provider or system. These dimensions are grouped into five As of access to care: affordability, availability, accessibility, accommodation, and acceptability [2]. Accessibility is ascertained by the geographic accessibility, and defined as how easily the client can physically reach the provider’s location, in other words, accessibility refers to the distance and time traveled to reach the facility [3,4]. Optimal access to healthcare services is essential for establishing a high quality of life in every community; however, equal access to healthcare services can be difficult to achieve due to multiple impediments. These impediments include but are not limited to a lack of awareness to the spatial distribution of healthcare services, challenging opportunities for private healthcare investors, and inadequate understanding of geographic variations and the saturation of the population. 

The prevalence of dental diseases is significantly high in the Kingdom of Saudi Arabia (KSA). The prevalence of dental caries among Saudi children aged 5–7 years and 12–15 years was estimated at 84% and 72%, respectively [5]. Alhabdan et al. [6] reported that the prevalence of dental caries among children in Riyadh was 83% (95% CI: 79.7–86.0%) [6]. Disease prevention at multiple levels is critical and many oral and dental diseases may be screened and treated during the early stages to hamper progression and further complications. Although dental caries is a disease that can be prevented easily, a lack of accessibility may contribute to its increasing prevalence. Enhancing accessibility to dental care services may encourage regular screening, aiding in early detection and more efficient management. Also, ease of access to dental care services in Riyadh may increase patients’ awareness and enhance their knowledge about the risk factors associated with dental caries and other oral diseases, hence, reducing the risk of developing such diseases.

To address accessibility challenges to dental care, a thorough understanding of the distribution of dental healthcare facilities is needed. Mapping the distribution of all facilities in Riyadh city in relation to the population density may help in illustrating saturated areas and thus guide authorities to target areas that are in greater need of dental care services. Moreover, it may benefit future dentists and investors planning to establish new practices by directing attention to the unserved/underserved areas in Riyadh to meet demands with less competition.

Previous studies reported some insightful information regarding the distribution of dental care facilities around the world. A study conducted in Japan found that even though there has been a significant improvement in the distribution of dental care facilities over the past years, 21% of municipalities with a population of 5000 or fewer still lack dental clinics [7]. More relevantly, studies conducted in Al Madina city and the Jazan region were consistent in finding that accessibility to dental care is challenging, particularly in rural areas [8,9,10]. In 2016, Alsalleeh and his co-workers [11] conducted an interesting general analysis of private dental clinics in Riyadh city and reported that private dental clinics were not evenly distributed in Riyadh city; however, they only determined the number of clinics in each sub-municipality without mapping out the detailed spatial distribution. Nonetheless, there are insufficient studies in the literature that address the spatial distribution of dental healthcare facilities in KSA and a gap needs to be filled specifically in Riyadh city. Therefore, this study aimed to evaluate the spatial distribution of PDHFs in Riyadh city, determine the saturation of PDHFs using PDHFs to population ratios, and investigate correlations between the number of PDHFs and other variables, such as area, total population, and mean monthly individual income. This makes the study the first of its kind in Riyadh city and may help in shedding light on possible gaps and disparities and point out overserved/underserved areas, thus benefiting health authorities and investors to target underserved or less saturated areas in Riyadh city for the better well-being of the residents.

## 2. Materials and Methods

Data collection for this study was conducted in August 2023. The study proposal was exempted from Institutional Review Board (IRB) review since it did not include human subjects or relevant data. Also, the data used were anonymous. Official approval was obtained from the research office at King Abdullah International Medical Research Center (#RSS23R/015). Riyadh city is the capital and highest populated city in KSA; it covers an area of 1973 km^2^ and its municipality includes 16 sub-municipalities [12]. This study analyzed the spatial distribution of private dental healthcare facilities (PDHFs) within these sub-municipalities. Moreover, the districts within these sub-municipalities were analyzed individually to calculate PDHFs per person.

### 2.1. Location of PDHFs

The current information regarding the number, location, and operability of PDHFs in Riyadh city was obtained from the Ministry of Health (MOH). All operating PDHFs were included: dental clinics, dental and dermatology polyclinics, and dental care departments located in hospitals. Whereas dental schools, mobile dental clinics, and government healthcare facilities were excluded. A total of 632 PDHFs were included in this study excluding 98 non-operating facilities or those with expired licensure. 

### 2.2. Geographic Integration

Ain el Abd/UTM zone 38N was used for coordinate referencing. The precise geographic location (GPS coordinates) of each sub-municipality and district was exported from the Riyadh region municipality website map [12] to Quantum Geographic Information System (QGIS) software (version 3.32.1, Essen, Germany), which was used to visualize, analyze, and interpret the geographic data. 

### 2.3. Mapping

All residential (15) sub-municipalities were included and analyzed in this study. The location of each PDHF was converted to longitude and latitude using Google Earth (version 10.52.0.0, Google, Sunnyvale, CA, USA) [13]. Then, each PDHF location was plotted on QGIS and treated as a central point. Four levels (radii) of buffer zones were determined as the covering range around each central point, with the difference between the levels being the distance from the central point. In other words, four levels of buffer zones—1 km, 3 km, 5 km, and >5 km—were determined to analyze the coverage of each private dental healthcare facility. The coverage of each facility was based on the area covered in square kilometers (km^2^).

### 2.4. Population Statistics

The population statistics and mean monthly individual income per district were gathered from the Zadd.910ths website, which is a governmental website that provides an interactive map with updated statistics [14].

### 2.5. Statistical Analysis

The area of each district and sub-municipality was calculated in square kilometers (km^2^) using QGIS. Descriptive statistics were determined using Microsoft Excel (version 16.0, Microsoft, Redmond, WA, USA). Pearson’s correlation test was performed to evaluate the strength of the association between continuous variables. RStudio software (version 4.1.3, Posit Software, PBC, Boston, MA, USA) was used to create the Lorenz curve. 

## 3. Results

**Figure 1:**
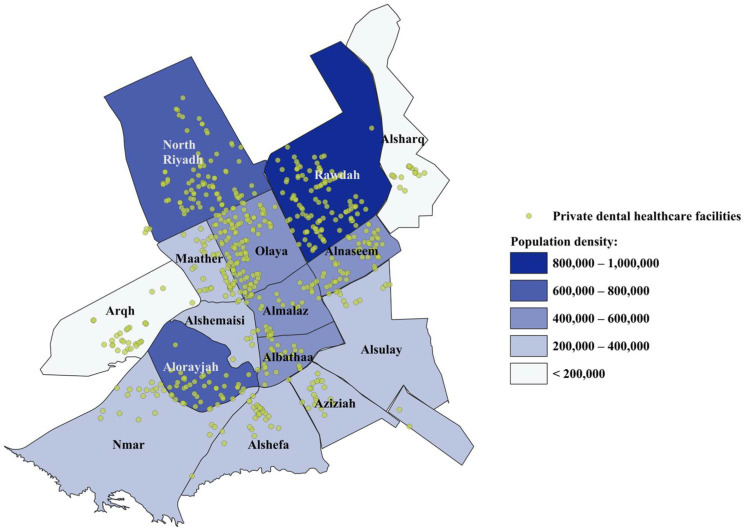
Population density and private dental healthcare facilities’ spatial distribution in Riyadh city.

**Figure 2:**
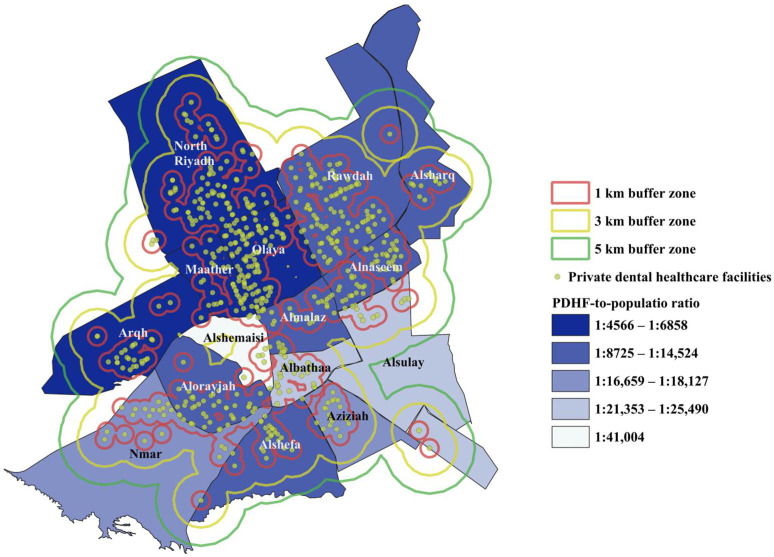
Coverage of private dental healthcare facilities and population ratios per sub-municipality in Riyadh city.

Riyadh city consists of 16 sub-municipalities with a population of 6,500,000 residents [14]. More than a third of these residents (35.4%, 2,225,665) reside mainly in three sub-municipalities: Rawdah (15.4%, 968,565), Alorayjah (10.4%, 653,600), and North Riyadh (9.6%, 603,500) (Table 1). A total of 632 PDHFs were geocoded by QGIS. Graphical data representation was used to show the distribution of PDHFs in Riyadh city. Figure 1 shows the population densities and private dental healthcare facilities’ spatial distribution in Riyadh city. Coverage areas of the dental facilities were mapped out and delineated by four buffer zones: 1 km, 3 km, 5 km, and >5 km, with each PDHF being a centroid (Figure 2). 

Almost half of the PDHFs (48.3%, 305/632) were clustered in only three sub-municipalities: Rawdah (17.6%, 111/632), Olaya (16.8%, 106/632), and North Riyadh (13.9%, 88/632); however, these three sub-municipalities have about one-third of total Riyadh population (32.6%, 2,056,028/6,500,000). Furthermore, although Olaya had the second-highest number of PDHFs, it was not one of the most populated sub-municipalities, ranked fifth regarding total population. North Riyadh and Alorayjah had similar total populations; however, the number of PDHFs in North Riyadh (88) was almost twice of that located in Alorayjah (45) (Table 1). On the other hand, Alshemaisi (1.1%, 7/632), Alsulay (1.9%, 12/632), and Alsharq (2.4%, 15/632) sub-municipalities had the lowest numbers of PDHFs. Looking at the districts’ level, Thrh Labn (3.79%, 24/632), Olaya (3.16%, 20/632), and Alnaseem Algharbi (3%, 19/632) districts had the highest numbers of PDHFs while there were 42 districts that did not have any PDHFs, mostly located in Albathaa (10 districts), Alshemaisi (8 districts), and Alsulay (5 districts) sub-municipalities (Table 1). 

**Figure 3:**
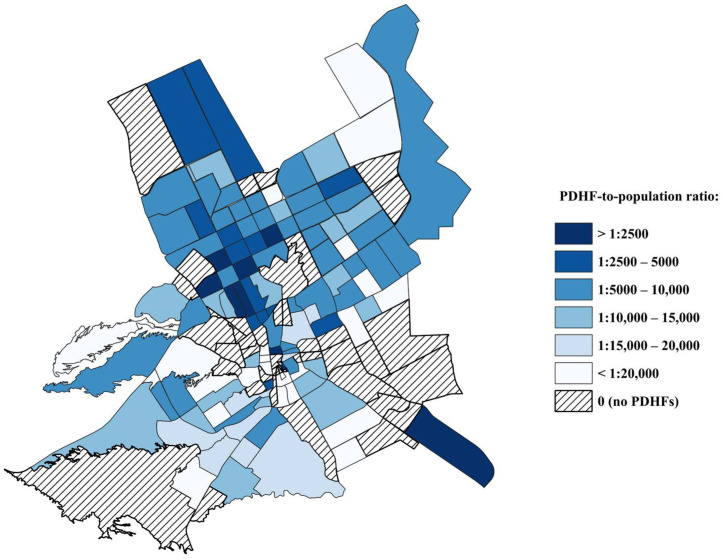
Private dental healthcare facilities-to-population ratios per district in Riyadh city.

The overall private dental healthcare facility-to-population ratio in Riyadh city was one PDHF per 9958 residents. Looking at the ratios of each sub-municipality, Olaya and Maather sub-municipalities had the largest PDHF-to-population ratios: (1:4566) and (1:4828), respectively. This may indicate the oversaturation of PDHFs in these two sub-municipalities as Olaya was not the most populated sub-municipality and Maather was the third least populated. On the contrary, Alshemaisi had the smallest PDHF-to-population ratio (1:41,004), followed by Alsulay (1:25,490) and Albathaa (1:21,353) (Table 1, Figure 2 and Figure 3). 

**Figure 4:**
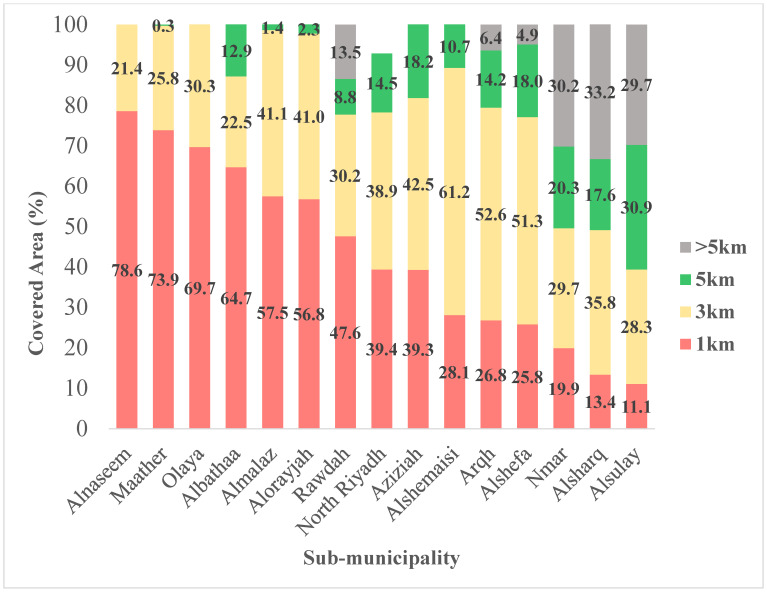
Comparison between the percentages of areas covered within four buffer zones for each sub-municipality in Riyadh city.

Looking at the covered areas in square kilometers (km^2^) using the four buffer zones around each PDHF, the majority of (86.7%) of Riyadh city total area was located within 5 km of a PDHF, and almost three-quarters (72.5%) of the total area was within 3 km while only a little above one third (36.3%) was within 1 km buffer zones. The sub-municipalities that had the highest coverage within 1 km buffer zones were Alnaseem (78.6%), Maather (73.9%), and Olaya (69.7%). All the districts of the Alnaseem sub-municipality had at least half (50%) of the area located within 1 km from a PDHF, with the Alrawabi district having complete (100%) coverage within 1 km (Figure 4). Moreover, the districts with the highest coverage in the Maather sub-municipality were Olaya and Almuhamadeyah, with complete (100%) coverage within 1 km buffer zones, while other districts had at least 50% coverage within 1 km buffer zones except for the King Saud University area (42.6%). Half of the districts located in the Olaya sub-municipality (7/14) were completely (100%) covered within 1 km buffer zones: Altaawuon, Olaya, Almaseef, Alworood, King Fahad, Alnuzhah, and Almorouj districts, while the remaining districts had at least 60% coverage within 1 km buffer zones except for the King Abdulaziz district (8.1%) that had the least coverage, followed by the King Abdullah district (44.9%). On the other hand, some sub-municipalities were underserved in Riyadh city. The sub-municipalities that had the least coverage within the 1 km buffer zones were Alsulay (11.1%), Alsharq (13.4%), and Nmar (19.9%). Moreover, these three sub-municipalities had the largest percentages of areas located out of 5 km buffer zones: Alsharq (33.2%), Nmar (30.2%), and Alsulay (29.7%) (Figure 4).

**Figure 5:**
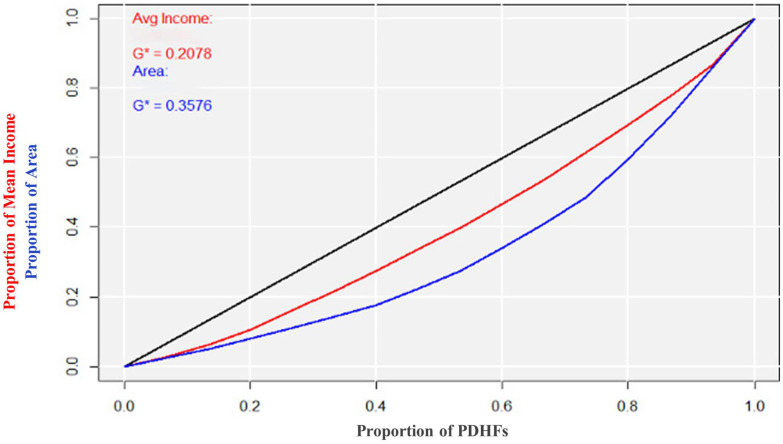
Lorenz curve and Gini coefficients: Riyadh city distribution of private dental healthcare facilities corresponding to mean individual income and area. PDHFs: private dental healthcare facilities. Avg: average (mean). G*: standardized Gini coefficient.

Pearson’s test showed an overall weak positive correlation between the number of PDHFs and total area of each sub-municipality (r = 0.29). However, there was a stronger overall positive correlation between the number of PDHFs and the total population in each sub-municipality (r = 0.74), indicating an increase in the number of PDHFs as the population increases; however, this does not necessarily mean the increase was even or followed a consistent proportion. Furthermore, the correlation between the mean individual income and number of PDHFs per district was negative in nine sub-municipalities: Alnaseem (r = −0.90), Alsharq (r = −0.72), Arqh (r = −0.47), North Riyadh (r = −0.44), Alshemaisi (r = −0.44), Olaya (r = −0.25), Nmar (r = −0.21), Maather (r = −0.16), and Rawdah (r = −0.05), while it was positive in the other six sub-municipalities: Aziziah (r = 0.69), Alsulay (r = 0.51), Albathaa (r = 0.38), Alorayjah (r = 0.23), Almalaz (r = 0.23), and Alshefa (r = 0.22). The Lorenz curve and Gini coefficient were determined to evaluate the distribution of PDHFs. The density of PDHFs corresponding to the area in square kilometers (km^2^) was estimated. The Lorenz curve was plotted with the cumulative proportion of PDHFs on the x-axis and proportions of mean income and area on the y-axis (Figure 5). The curve in blue color represents the distribution of PDHFs in Riyadh city corresponding to the area with a Gini coefficient of 0.357, indicating a moderate level of disparity in the distribution of PDHFs corresponding to the area. The curve in red color represents the distribution of PDHFs corresponding to the mean income with a Gini coefficient of 0.207, indicating almost equality in the distribution of PDHFs in Riyadh city corresponding to the mean income. 

## 4. Discussion

Accessibility to dental healthcare is a key factor affecting the utilization of dental services, in addition to financial reasons, attitudes toward dental care [15], and the availability of high-quality dental services [16,17]. Enhancing access to healthcare is one of the goals of the Saudi Vision 2030, and the private healthcare sector is an essential partner to help in achieving this important goal. The dental healthcare private sector in Riyadh city has been growing rapidly over the past few years. However, there is a lack of information regarding the spatial distribution of PDHFs and a need for a more comprehensive analysis and details about the saturation of PDHFs. The distribution of PDHFs in Riyadh city is an important topic as it sheds light on the accessibility to dental healthcare services. Therefore, this study was conducted.

As of August 2023, there were 632 operating PDHFs registered in MOH. A Geographical Information System (GIS)-based approach was utilized in this study. The project started with analyzing the map of Riyadh city, its sub-municipalities, and districts. According to the governmental website of Riyadh Municipality Portal, Riyadh city is divided into 16 sub-municipalities. Fifteen sub-municipalities were included and analyzed in this study [12]. Alhayer sub-municipality was excluded due to the lack of population statistics and the absence of registered PDHFs, which could be explained by the fact that it included mainly non-residential areas.

Each PDHF was treated as a central point and buffer zones were created around each one. Four levels of buffer zones were selected for this study with 1 km being the smallest as shown in Figure 2. One kilometer represents the shortest straight-line distance (radius) around each PDHF and may not represent the actual route, which would likely be longer driving a car considering road obstacles and traffic. In addition, Riyadh is a metropolitan area and the capital city with significant traffic congestion (traffic congestion index = 21.98) [18]. There is no universally approved distance for access to care. Riyadh city is considered the most congested city in Saudi Arabia with a peak vehicle speed of 46.7 km/h during morning and afternoon periods [19]. Thus, the levels of buffer zones were set at 1 km, 3 km, 5 km, taking into consideration the time to travel to the nearest dental healthcare facility in each district and the relative saturation of PDHFs in Riyadh. Several sub-municipalities in Riyadh city showed multiple shared patterns of PDHFs distribution and PDHF-to-population ratios. For instance, Olaya, Maather, North Riyadh, and Rawdah sub-municipalities had the largest PDHF-to-population ratios at 1:4566, 1:4828, 1:6858, 1:8726, respectively. The percentages of the areas covered within 1 km buffer zones were Maather (73.9%), Olaya (69.7%), Rawdah (47.6%), and North Riyadh (39.4%). A total of 347 PDHFs (54.9% of included PDHFs) were located within these four sub-municipalities. This may indicate significant clustering of PDHFs in these sub-municipalities. On the other hand, less than 50% of the areas of Alshefa, Alsharq, Nmar, Aziziah, and Alsulay sub-municipalities were within 1 km from 92 facilities (Figure 4). The PDHF-to-population ratios in these sub-municipalities were 1:11,924, 1:12,289, 1:16,659, 1:18,127, and 1:25,490.25, respectively. This may demonstrate an uneven distribution of PDHFs with an inadequate number of facilities to serve these highly populated sub-municipalities. In Alnaseem, Almalaz, Alorayjah, and Albathaa sub-municipalities, there were 157 PDHFs distributed over 51 districts, with more than 50% of the area of these sub-municipalities being within 1 km of PDHFs (Figure 4). However, the PDHF-to-population ratios in these sub-municipalities were relatively low, precisely equal to 1:10,208, 1:11,102, 1:14,524, and 1:21,353, respectively, indicating the number of PDHFs might not be sufficient to serve the entire population in these sub-municipalities although the spatial distribution of PDHFs seemed relatively even (Figure 2). A more unique pattern of distribution was noticed in two sub-municipalities: Arqh and Alshemaisi. Arqh sub-municipality had the third largest PDHF-to-population ratio (1:6011); however, only 26.8% of the total areas were within 1 km of 29 PDHFs. This may demonstrate that although the number of PDHFs seemed quite adequate to serve the population, the facilities were not evenly distributed across these two sub-municipalities. In contrast, Alshemaisi sub-municipality had only seven PDHFs with the lowest PDHF-to-population ratio (1:41,004), and only 28.1% of the area was within a 1 km range of PDHFs, indicating a lack of private facilities in this sub-municipality. This could be explained by the presence of a large governmental healthcare facility, King Saud Medical City. In general, the findings of the present study showed a tendency of significant clustering of PDHFs in northern, central, and northeastern parts of Riyadh city.

Previous studies were conducted to analyze the distribution of dental services in KSA. Shubayr and his co-workers [10] reported that there was an uneven distribution of dental services in the Jazan region, although there was a positive correlation between the distribution of dental clinics and population density across Jazan region, which is in agreement with the findings of this study. The overall dental clinics-to-population ratio reported in the Jazan region was one PDHF per 6279 residents, which is more favorable than the ratio found in the present study (1:9958). This could be explained by the fact that Riyadh city is more densely populated, being the capital city, and that Shubayr and his co-workers included both public and private dental clinics in their analysis. On the contrary, a study conducted in 2020 reported a ratio of one dental clinic per 13,647 residents in Al Madina city, which is much lower although both public and private dental clinics were included [8]. A study conducted in 2016 reported that the overall dental clinic-to-population ratio in Malaysia was 1:9000; 1:38,000 for public dental clinics and 1:13,000 for private facilities; however, the clinics were unevenly distributed across the country [20].

Alsalleeh and his co-workers [11] investigated the number of private dental clinics and the number of dentists in Riyadh city. They reported that private dental clinics were not evenly distributed in Riyadh city, which is in agreement with the present study. However, they determined the number of clinics in each sub-municipality without mapping out the exact distribution. Mapping the clinics in GIS should give further details regarding the facilities’ distribution, saturation, and accessibility in each district. The number of dental clinics included in their study was obtained from the Ministry of Health (MOH)—a total of 236 registered dental clinics. Comparing the number of dental clinics registered in 2016 to the number collected in this study shows that the number of dental healthcare facilities has tripled, signifying the rapid expansion of this sector over the past few years. Their results showed that Olaya and Rawdha had the highest number of dental clinics, 49 and 45, respectively, which aligns with the results found in the present study, 106 and 111, respectively. Alsalleeh and his co-workers collected information about the number of dentists and available specialties; however, almost one third of (31.4%, 74/236) the dental clinics refused to be enrolled.

The Lorenz curve and Gini coefficient are essential tools in economics that provide insight into income distribution within a population. The Lorenz curve is a graphical representation of income distribution, plotting the cumulative percentage of income against the cumulative percentage of the population [21]. In an ideal egalitarian society, the Lorenz curve would be a diagonal line indicating perfect income equality. However, in reality, it usually curves away from the diagonal line, reflecting varying degrees of income inequality. The Gini coefficient, derived from the Lorenz curve, is a numerical measure that quantifies this inequality which was introduced by the Italian statistician Corrado Gini in his work ‘*Variability and Mutability*’ published in Italian in 1912 [22]. It ranges from 0 to 1, where 0 indicates perfect equality, and 1 indicates extreme inequality. The current study used a similar concept to explain the geographical distribution of PDHFs in Riyadh city and showed that there was a moderate level of disparity in the distribution of PDHFs corresponding to the area (Gini coefficient = 0.357). To further illustrate this, as the number of PDHFs increases, the Lorenz curve shifts towards the upper-right portion of the graph. In other words, a greater proportion of PDHFs is concentrated in a smaller proportion of the area as the number of PDHFs increases. However, there was almost equality in the distribution of PDHFs in Riyadh city corresponding to the mean income (Gini coefficient = 0.207). In other words, as the number of PDHFs increases, the Lorenz curve shifts towards the upper-left portion of the graph. This movement signifies an improvement in the mean income according to the increase in the number of PDHFs.

This study is the first to evaluate the distribution of private dental healthcare facilities in Riyadh city using GIS and showed significant findings. However, in order to achieve optimal access to healthcare services, multiple factors in addition to the spatial distribution of PDHFs need to be analyzed, such as the actual number of dental chairs per facility, number of dentists and dental auxiliaries or clinicians’ full time equivalent (FTE) per facility, and the available dental specialties and types of services provided. In other words, the number of PDHFs may not accurately represent the exact capacity (number of dental chairs or dentists); therefore, obtaining additional information about the actual number of dental chairs and dental staff and analyzing it with regard to the spatial distribution is recommended for future studies, which may help in reaching a more detailed view of the supply and demand in dental health care services. The findings of the present study established a foundation for the spatial distribution of PDHFs in Riyadh city. One of the limitations of this study is that it did not include the governmental dental healthcare facilities, which are considered a significant sector in providing healthcare services in Saudi Arabia in general and Riyadh city in particular. It would be beneficial to also map out and integrate the governmental facilities in Riyadh city to attain a more comprehensive perspective. Another limitation is having data collected from a single point in time. In other words, Riyadh city is rapidly developing, and new dental healthcare facilities are continually being established.

## 5. Conclusions

There was an uneven distribution of PDHFs in Riyadh city. Some areas were underserved while others were overserved in several sub-municipalities. This highlights the need for improving the distribution and accessibility of dental healthcare facilities in Riyadh city. It is fundamental to ensure that residents have optimal access to care. Policy-makers and investors are encouraged to target underserved areas rather than areas with significant clustering to improve access and quality of care.

## Figures and Tables

**Table 1:** Private dental healthcare facilities-to-population ratios per sub-municipality in Riyadh city.

Sub-Municipality	PDHF (No.)	Population (No.)	PDHF-to-Population Ratio
**Olaya**	106	483,963	1:4566
**Maather**	42	202,782	1:4828
**Arqh**	29	174,305	1:6011
**North Riyadh**	88	603,500	1:6858
**Rawdah**	111	968,565	1:8725
**Alnaseem**	52	530,800	1:10,208
**Almalaz**	37	410,776	1:11,102
**Alshefa**	24	286,173	1:11,924
**Alsharq**	15	184,328	1:12,289
**Alorayjah**	45	653,600	1:14,524
**Nmar**	22	366,500	1:16,659
**Aziziah**	19	344,415	1:18,127
**Albathaa**	23	491,119	1:21,353
**Alsulay**	12	305,883	1:25,490
**Alshemaisi**	7	287,027	1:41,004
**Total**	632	6,293,736	1:9958

**PDHF:** Private dental healthcare facility.

## Data Availability

Most of the data supporting our findings is contained within the manuscript, and all others will be shared upon request.

## Abbreviations

GIS	Geographical Information System
PDHFs	Private dental healthcare facilities
KSA	Kingdom of Saudi Arabia
MOH	Ministry of Health
QGIS	Quantum Geographic Information System
km^2^	Square kilometers
FTE	Full time equivalent
MNGHA	Ministry of National Guard-Health Affairs
KAIMRC	King Abdullah International Medical Research Center.
IRB	Institutional Review Board

## References

[1] McLafferty S.L. (2003). GIS and Health Care. Annu. Rev. Public Health..

[2] Penchansky R., Thomas J.W. (1981). The concept of access: Definition and relationship to consumer satisfaction. Med. Care.

[3] Apparicio P., Abdelmajid M., Riva M., Shearmur R. (2008). Comparing alternative approaches to measuring the geographical accessibility of urban health services: Distance types and aggregation-error issues. Int. J. Health Geogr..

[4] Bullen N., Moon G., Jones K. (1996). Defining localities for health planning: A GIS approach. Soc. Sci. Med..

[5] Adam T.R., Al-Sharif A.I., Tonouhewa A., AlKheraif A.A. (2022). Prevalence of Caries among School Children in Saudi Arabia: A Meta-Analysis. Adv. Prev. Med..

[6] Alhabdan Y.A., Albeshr A.G., Yenugadhati N., Jradi H. (2018). Prevalence of dental caries and associated factors among primary school children: A population-based cross-sectional study in Riyadh, Saudi Arabia. Environ. Health Prev. Med..

[7] Okawa Y. (2014). Trends in the geographic distribution of dental clinics in Japan. Community Dent. Health.

[8] Alsharif A.T. (2020). Georeferencing of Current Dental Service Locations to Population Census Data: Identification of Underserved Areas in Al Madina, Saudi Arabia. Sage Open.

[9] Shubayr M.A., Kruger E., Tennant M. (2022). Geographic accessibility to public dental practices in the Jazan region of Saudi Arabia. BMC Oral. Health.

[10] Shubayr M.A., Kruger E., Majeed M.M., Hattan A.H., Jearan S.A., Tennant M. (2023). Distribution of dental practices in Jazan of Saudi Arabia: A GIS-based approach. BMC Health Serv. Res..

[11] Alsalleeh F., Alohali M., Alzeer M., Aloseimi M., Almuflehi N., Alshiha S. (2018). Analyzing private dental clinics in Riyadh City, Saudi Arabia. Saudi Dent. J..

[12] Riyadh Region Municipality Published August 2023. https://www.alriyadh.gov.sa/ar/Municipalities.

[13] Google Earth Published Online 2023. https://earth.google.com/web/.

[14] Zadd.910ths. Published 2019. https://zadd.910ths.sa/ar.

[15] Al-Hussyeen A.J.A. (2010). Factors affecting utilization of dental health services and satisfaction among adolescent females in Riyadh City. Saudi Dent. J..

[16] Saeed A.A., Mohamed B.A. (2002). Patients’ perspective on factors affecting utilization of primary health care centers in Riyadh, Saudi Arabia. Saudi Med. J..

[17] Butters J.M., Willis D.O. (2000). A comparison of patient satisfaction among current and former dental school patients. J. Dent. Educ..

[18] TrafficIndex.org. Riyadh Traffic Congestion Report. Published 2024. https://trafficindex.org/riyadh/.

[19] INRIX Published 2023. https://inrix.com/scorecard-city-2023/?city=Riyadh&index=165.

[20] Md Bohari N.F., Kruger E., John J., Tennant M. (2019). Analysis of dental services distribution in Malaysia: A geographic information systems–based approach. Int. Dent. J..

[21] Lorenz M.O. (1905). Methods of Measuring the Concentration of Wealth. Publ. Am. Stat. Assoc..

[22] Gini C. (1912). Variabilità e Mutabilità: Contributo Allo Studio Delle Distribuzioni e Delle Relazioni Statistiche [Fasc. I.].

